# Viable and Functional: Long-Term −80 °C Cryopreservation Sustains CD34^+^ Integrity and Transplant Success

**DOI:** 10.3390/jcm14197032

**Published:** 2025-10-04

**Authors:** Ibrahim Ethem Pinar, Muge Sahin, Vildan Gursoy, Tuba Ersal, Ferah Budak, Vildan Ozkocaman, Fahir Ozkalemkas

**Affiliations:** 1 Department of Hematology, Bursa Uludag University Faculty of Medicine, 16059 Bursa, Turkey; vildanterzioglu@hotmail.com (V.G.); tubaersal@uludag.edu.tr (T.E.); vildanoz@uludag.edu.tr (V.O.); fahir@uludag.edu.tr (F.O.); 2 Department of Internal Medicine, Bursa Uludag University Faculty of Medicine, 16059 Bursa, Turkey; muge.sahin.ms@gmail.com; 3 Department of Immunology, Bursa Uludag University Faculty of Medicine, 16059 Bursa, Turkey; fbudak@uludag.edu.tr

**Keywords:** cryopreservation, CD34^+^ viability, hematopoietic stem cell transplantation, −80 °C storage, engraftment, flow cytometry

## Abstract

**Background:** Cryopreservation of hematopoietic stem cells (HSCs) at −80 °C using uncontrolled-rate freezing is frequently employed in resource-constrained settings, yet concerns remain regarding long-term viability and clinical efficacy. Reliable post-thaw assessment is essential to ensure graft quality and engraftment success. **Methods:** This single-center, retrospective study evaluated 72 cryopreserved stem cell products from 25 patients stored at −80 °C for a median of 868 days. Viability was assessed using both acridine orange (AO) staining and 7-AAD (7-aminoactinomycin D) flow cytometry at three time points: collection (T0), pre-infusion (T1), and delayed post-thaw evaluation (T2). Associations between viability loss, storage duration, and clinical engraftment outcomes were analyzed. **Results:** Median post-thaw viability remained high (94.8%) despite a moderate time-dependent decline (~1.02% per 100 days; R^2^ = 0.283, *p* < 0.001). Mean viability loss at T2 was 9.2% (AO) and 6.6% (flow cytometry). AO demonstrated greater sensitivity to delayed degradation, with a significant difference between methods (*p* < 0.001). Engraftment kinetics were preserved in most patients, with neutrophil and platelet recovery primarily influenced by disease type rather than product integrity. Notably, storage duration and donor age were not significantly associated with engraftment outcomes or CD34^+^ cell dose. **Conclusions:** Long-term cryopreservation at −80 °C maintains HSC viability sufficient for durable engraftment, despite gradual decline. While transplant outcomes are primarily dictated by disease biology and remission status, AO staining provides enhanced sensitivity for detecting delayed cellular damage. Notably, our viability-loss model offers a practical framework for predicting product quality, potentially supporting graft selection and clinical decision-making in real-world, resource-constrained transplant settings.

## 1. Introduction

The cryopreservation of hematopoietic stem cells (HSCs) is a critical enabler of modern transplantation, providing logistical flexibility and supporting both autologous and allogeneic procedures. However, concerns persist regarding the impact of prolonged storage—especially under −80 °C conditions and with uncontrolled-rate freezing—on the viability and functional integrity of stored grafts. A reliable assessment of post-thaw viability is essential for ensuring product quality and predicting clinical outcomes [1,2,3].

Multiple techniques have been employed to evaluate cell viability following thawing [4,5]. Acridine orange (AO) staining and flow cytometry with 7-Aminoactinomycin D (7-AAD) or viability dyes are among the most widely used. Prior studies have shown good concordance between these approaches, with AO-based staining offering a rapid, non-toxic alternative that may retain stability even in delayed post-thaw assessments [4,6]. More recent evaluations have confirmed that both methods yield comparable results, with flow cytometry-based 7-AAD assays showing a strong correlation with acridine orange/ethidium bromide (AO/EB) microscopy, and both proving reliable for assessing viability in clinical peripheral blood stem cell products [7,8].

Nevertheless, viability losses remain highly variable, influenced by cryopreservation conditions, pre-freezing cell handling, and storage duration. Evidence suggests that uncontrolled freezing at −80 °C may result in greater viability loss compared to standard cryopreservation in liquid nitrogen. Donmez et al. [9] reported a mean CD34^+^ cell viability loss of 48.5% following uncontrolled-rate freezing at −80 °C, and similarly, Grommé [10] observed markedly reduced viability in products that were stored overnight at 2–8 °C prior to cryopreservation. In contrast, other studies have shown preserved hematopoietic potential even after more than 5–10 years of cryostorage in vapor-phase nitrogen [11,12].

Viability and CD34^+^ cell dose at infusion are both linked to engraftment kinetics and clinical recovery. While lower post-thaw viability has been associated with an increased risk of complications, some reports indicate that long-term cryopreserved products can still support robust engraftment [13,14]. Moreover, recent efforts to enhance graft quality have extended beyond post-thaw assessments; notably, Alswied et al. developed large-scale machine learning models using data from over 17,000 healthy donors, demonstrating superior predictive accuracy in estimating CD34^+^ cell yield prior to collection and thereby enabling more individualized apheresis planning [15]. These discrepancies underscore the need for systematic evaluation of product quality in real-world settings.

In this single-center study, we aimed to address these gaps by systematically evaluating the long-term viability and functional adequacy of CD34^+^ progenitor cell products stored at −80 °C. Using both AO staining and 7-AAD flow cytometry, we compared cell viability across three time points: initial collection, pre-infusion, and delayed post-thaw assessment. We further explored correlations between viability loss and clinical outcomes—including engraftment kinetics, storage duration, and transplantation success—to determine whether prolonged storage under suboptimal conditions compromises product efficacy.

## 2. Methods

### 2.1. Study Design and Ethics

This was a single-center, retrospective observational study involving adult patients who underwent CD34^+^ progenitor cell collection and/or transplantation at our institution between December 2016 and September 2020. Stem cell products were cryopreserved and stored at −80 °C in the institutional biobank. All included patients were deceased at the time of ethical approval. The study protocol was approved by the local ethics committee prior to the scheduled disposal of stored products, and viability analyses were prospectively performed on the cryopreserved units prior to destruction in accordance with institutional biobank regulations (Ethics approval number: 2021-7/49, Date: 2 June 2021)

### 2.2. Study Population

A total of 25 adult patients who underwent HSC mobilization and/or transplantation at our institution between December 2016 and September 2020 were included. Demographic and baseline clinical data were retrieved from electronic medical records.

In total, 72 cryopreserved products were available for long-term viability analysis. These did not represent multiple mobilization courses in the same patient; rather, in accordance with institutional practice, each leukapheresis collection—including those performed on consecutive days when required—was divided into multiple cryopreserved products. This explains the higher number of stored units relative to the number of mobilized patients.

Among 20 patients who required only a single leukapheresis session, 46 products were generated (median 2 per patient, range 1–6). In the remaining 5 patients, leukapheresis was performed on two consecutive days, yielding 26 products (median 5 per patient, range 3–8). Products from different days were stored separately and not pooled.

Although viability was assessed in 72 products, paired AO/EB and 7-AAD data were available for 69. In three cases, parallel assessments could not be performed due to insufficient residual volume or technical limitations, and these were excluded from correlation analysis.

The cryopreserved products included in this analysis were not transplanted. They had been stored for potential second transplantation or donor lymphocyte infusion, but the intended recipients died before use. For patients who underwent transplantation, infused grafts and corresponding CD34^+^ cell dose data were obtained retrospectively from medical records.

### 2.3. Stem Cell Collection and Processing

Mobilization strategies included G-CSF alone, G-CSF plus chemotherapy, or G-CSF plus plerixafor, and in selected cases, unstimulated bone marrow harvest. For G-CSF–only mobilization, patients received filgrastim (Neupogen^®^, Amgen, Thousand Oaks, CA, USA) 10 µg/kg/day subcutaneously for 5 consecutive days; leukapheresis was initiated two hours after the final dose. In chemotherapy-based mobilization, G-CSF at 10 µg/kg/day was started following chemotherapy, and leukapheresis was performed once peripheral CD34^+^ counts exceeded 10–20/µL, typically between days 8 and 12. For plerixafor-based mobilization, patients received G-CSF for 4 days, followed by plerixafor (Mozobil^®^, Sanofi, Bridgewater, NJ, USA) 0.24 mg/kg subcutaneously on the evening of day 4; leukapheresis was conducted the following morning. In selected cases, unstimulated bone marrow harvests were performed under general anesthesia from posterior iliac crests, with 10–15 mL/kg aspirated into heparinized bags, filtered, and processed under sterile conditions.

Leukapheresis was performed on the Spectra Optia^®^ Apheresis System (Terumo BCT, Lakewood, CO, USA), processing 100–150 mL/kg of blood at 40–50 mL/min, with an average duration of 3–4 h. Apheresis products were divided into multiple cryopreserved products as per routine practice. The target yield was ≥4 × 10^6^ CD34^+^ cells/kg recipient body weight. CD34^+^ cell enumeration in both peripheral blood stem cells and bone marrow products was performed using single-platform flow cytometry based on the International Society of Hematotherapy and Graft Engineering (ISHAGE) guidelines [16] and its single-platform modification [17], on a Navios EX cytometer (Beckman Coulter, Brea, CA, USA) using anti-CD34-FITC (clone 581, BD Biosciences), anti-CD45-PE (clone J.33, Beckman Coulter), and 7-AAD (BD Biosciences).

### 2.4. Conditioning Regimens and Transplantation

All patients who proceeded to transplantation received myeloablative conditioning tailored to diagnosis, comorbidities, and institutional practice. High-dose melphalan was used for multiple myeloma; busulfan–cyclophosphamide or etoposide–cyclophosphamide with total body irradiation were used for acute leukemias; and melphalan 140 mg/m^2^ was administered in older patients or those with renal impairment. Detailed distribution of regimens is provided in Table 1.

Stem cell products were thawed immediately prior to infusion in a 37 °C water bath (Memmert GmbH, Schwabach, Germany) with gentle agitation until ice-free. Products were infused via central venous catheter without washing to minimize cell loss and delays. All patients received premedication with antihistamines and corticosteroids and were monitored closely for infusion-related adverse events, particularly those associated with dimethyl sulfoxide (DMSO).

### 2.5. Viability Assessment

Viability of cryopreserved products was assessed using two complementary methods: AO/EB staining and 7-AAD flow cytometry with absolute counting. No CD34^+^ sorting or enrichment was performed; all assays were conducted on bulk leukapheresis products.

Cryopreserved products were thawed in a 37 °C water bath with gentle agitation until ice-free, diluted 1:10 in RPMI-1640 medium (Gibco, Thermo Fisher Scientific, Waltham, MA, USA) supplemented with 20% autologous plasma, and analyzed within 30 min to avoid post-thaw artifacts.

For AO/EB staining, a 20 µL aliquot was mixed 1:1 with AO/EB solution (Sigma-Aldrich, St. Louis, MO, USA) and examined under a Leica DMi8 fluorescence microscope (Leica Microsystems, Wetzlar, Germany). At least 200 nucleated cells were counted. Viability was expressed as the proportion of AO^+^/EB^−^ cells among total nucleated cells.

For 7-AAD flow cytometry, samples were stained with anti-CD34-FITC (clone 581, BD Biosciences, San Jose, CA, USA), anti-CD45-PE (clone J.33, Beckman Coulter, Brea, CA, USA), 7-AAD (7-AAD, BD Biosciences), and Flow-Count fluorospheres (Beckman Coulter, Brea, CA, USA). Acquisition was performed on a Navios EX cytometer (Beckman Coulter, Brea, CA, USA), with at least 100,000 events collected per tube and analyzed in Kaluza software version 2.1 (Beckman Coulter). Gating was performed on CD45^+^/SSC^low leukocytes, with CD34^+^ progenitors identified within this population. Viable CD34^+^ cells were defined as 7-AAD negative. Absolute viable CD34^+^ counts were calculated volumetrically; when direct post-thaw enumeration was not feasible, values were estimated by adjusting pre-thaw totals according to measured post-thaw viability.

Viability was assessed at up to three time points: T0 (fresh, baseline), T1 (day of transplantation, post-thaw), and T2 (long-term post-thaw). Viability loss was defined as the absolute percentage difference between T0–T1 (short-term loss) or T0–T2 (long-term loss). Results from AO/EB and 7-AAD methods were compared using correlation analyses.

### 2.6. Engraftment Definitions

Neutrophil engraftment was defined as the first of three consecutive days with an absolute neutrophil count ≥0.5 × 10^9^/L, confirmed by daily counts, in accordance with the European Society for Blood and Marrow Transplantation (EBMT) and the Center for International Blood and Marrow Transplant Research (CIBMTR) criteria [18,19]. Platelet engraftment was defined as the first of seven consecutive days with a platelet count ≥20 × 10^9^/L without transfusion. Sustained recovery to ≥50 × 10^9^/L without transfusion for at least seven days was also recorded.

### 2.7. Statistical Analysis

Continuous variables were summarized as median (range) and compared using the Mann–Whitney U test (two groups) or Kruskal–Wallis test (multiple groups). Categorical variables were expressed as counts (percentages) and compared using Fisher’s exact test. Correlations were assessed with Spearman’s ρ, and the association between storage duration and viability loss was tested with linear regression.

Overall survival (OS) was defined as the time from diagnosis to death from any cause, and progression-free survival (PFS) as the time from diagnosis to relapse/progression or death. Survival was estimated using Kaplan–Meier curves and compared with the log-rank test.

All analyses were two-sided, with *p* < 0.05 considered statistically significant. Statistical analysis was performed in Python (v3.13) using SciPy, Lifelines, and Statsmodels packages.

## 3. Results

### 3.1. Long-Term Cryostorage Preserves CD34^+^ Viability Despite Time-Dependent Decline

A total of 25 patients were included in the study, with a median age of 54 years (range, 19–63), and 60% were male. Multiple myeloma was the most common diagnosis (52%), followed by acute lymphoblastic leukemia (32%). G-CSF alone was the predominant mobilization regimen (76%). Stem cell transplantation was performed in 80% of patients, all of whom received myeloablative conditioning, most commonly Melphalan 200 mg/m^2^ (50%) (Table 1).

A total of 72 cryopreserved CD34^+^ progenitor cell products were analyzed. The median storage duration at −80 °C was 868 days (range, 382–1763). At initial collection (T0), the median total cell viability was 100% (range, 95.2–100), and CD34^+^ cell viability was similarly high (median 100%, range, 98.6–100). The median infused CD34^+^ cell dose, normalized to recipient body weight, was 8.5 × 10^6^ cells per kilogram. Products contained a median white blood cell count of 180.5 × 10^9^/L, including neutrophils (72.9 × 10^9^/L), lymphocytes (51.4 × 10^9^/L), and monocytes (27.9 × 10^9^/L), with a median mononuclear cell to total white blood cell ratio of 0.60 (range, 0.23–0.99) (Table 2).

**Figure 1:**
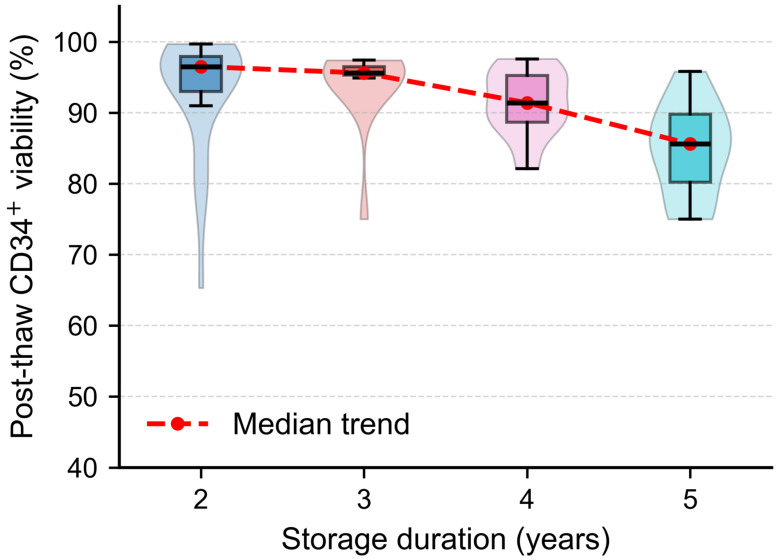
Post-thaw CD34^+^ viability by storage duration. Violin/box plots illustrating post-thaw CD34^+^ cell viability in cryopreserved products stratified by storage duration. Box plots indicate medians and interquartile ranges, while violin shapes depict distribution density. The red dashed line represents the median viability trend across storage years, demonstrating a gradual, time-dependent decline and increasing variability beyond 4 years.

Post-thaw CD34^+^ viability remained consistently high across all storage groups but demonstrated a gradual, time-dependent decline with longer cryostorage. Violin/box plots stratified by storage duration illustrate this trend, showing stable viability during the first 2–3 years, followed by progressive reductions in median values and increased variability beyond 4 years (Figure 1).

### 3.2. Engraftment Dynamics Reflect Disease Biology Rather than Product Integrity

Neutrophil engraftment (>0.5 × 10^9^/L) occurred at a median of 13.5 days (range, 7–23), while platelet engraftment > 50 × 10^9^/L was achieved at a median of 16.5 days (range, 13–37) (Table 3). Among patients who proceeded to transplantation, unmanipulated cryopreserved peripheral blood stem cell grafts were infused at a median CD34^+^ cell dose of 5.85 × 10^6^/kg (range 3.42–8.1 × 10^6^/kg), and engraftment outcomes were consistent with expected clinical standards. To further address differences between product types, we directly compared product and harvest characteristics in transplanted patients who received fresh versus frozen grafts. Except for a significantly higher harvest neutrophil count in the frozen group (*p* = 0.0326), no other statistically significant differences were observed (Table 4).

To complement viability percentages, absolute CD34^+^ counts were assessed at sequential time points. At initial collection (T0, pre-thaw), the total CD34^+^ yield was a median of 1770.5 cells/µL (range, 18–3591 cells/µL). On the day of transplantation (T1), post-thaw viable CD34^+^ counts were measured by both AO staining and flow cytometry, yielding medians of 2870.0 (range, 2330.1–3591.0 cells/µL) and 2400.4 (range, 1939.3–3458.1 cells/µL), respectively. For long-term cryopreserved products from deceased patients (T2), viable CD34^+^ counts remained within clinically relevant ranges, with medians of 1623.3 (range, 15.9–3447.4 cells/µL) by AO and 1573.7 (range, 17.5–3551.5 cells/µL) by flow cytometry.

**Figure 2:**
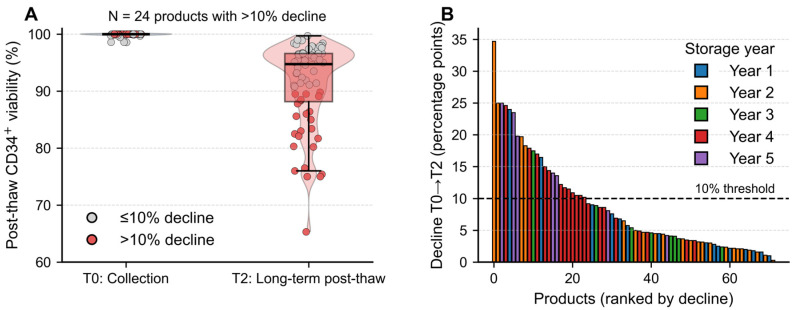
Visualization of post-thaw CD34^+^ viability decline in long-term cryopreserved products. (**A**) Violin/box/jitter plots showing CD34^+^ cell viability at collection (T0) and long-term post-thaw (T2). Individual products are displayed, with those showing ≤10% viability decline marked in grey and those with >10% decline highlighted in red (N = 24, 33.3%). The median post-thaw viability was 94.8%. (**B**) Waterfall plot ranking all 72 products according to the magnitude of viability decline (T0 → T2). Each bar represents a single product, color-coded by storage year. The dashed line denotes the 10% decline threshold, allowing immediate visualization of products exceeding this cutoff.

The median post-thaw viability (T2) was 94.8%, with 24 products (33.3%) showing a viability decline exceeding 10% compared to the initial collection (T0) (Figure 2). Importantly, none of these high-loss products had been stored for less than 200 days, reinforcing the interpretation that degradation was primarily time-dependent rather than procedural.

Subgroup analyses did not identify significant differences in CD34^+^ dose or engraftment kinetics based on sex or comorbidity (Table 3). However, patients with acute lymphoblastic leukemia demonstrated significantly longer neutropenia duration (median: 27 vs. 8.5 days, *p* < 0.001) and delayed neutrophil recovery (*p* < 0.001) compared to those with multiple myeloma.

Interestingly, patients not in complete remission at transplantation exhibited faster platelet engraftment at >20 × 10^9^/L (*p* = 0.012). Recipients of fresh products had significantly longer neutropenia duration (median 27 vs. 9 days, *p* = 0.0144) and also experienced delayed neutrophil engraftment (median 17 vs. 11.5 days, *p* = 0.0179), which likely reflects disease-specific transplant patterns rather than product quality alone.

Spearman correlation analysis showed no significant relationship between donor age and post-thaw viability (ρ = −0.087, *p* = 0.678), CD34^+^ cell viability (ρ = −0.181, *p* = 0.388), or total cell viability (ρ = 0.176, *p* = 0.585). Similarly, age was not significantly correlated with infused CD34^+^ cell dose (ρ = −0.342, *p* = 0.140), platelet engraftment > 20 × 10^9^/L (ρ = −0.022, *p* = 0.931), or >50 × 10^9^/L (ρ = 0.066, *p* = 0.808) (Table 5). Interestingly, younger age was associated with significantly prolonged neutropenia (ρ = −0.761, *p* < 0.001) and delayed neutrophil engraftment (ρ = −0.750, *p* < 0.001). This unexpected association likely reflects the underlying disease distribution, as younger patients in the cohort were more frequently diagnosed with acute leukemia, which is known to involve more intensive pretransplant treatments and delayed hematopoietic recovery.

**Figure 3:**
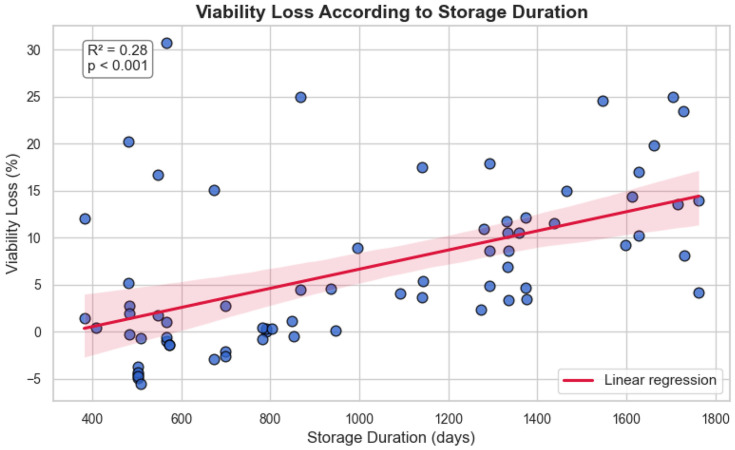
Association Between Storage Duration and Post-Thaw Viability Loss. Each blue dot represents an individual sample, plotted according to its storage duration (*x*-axis) and corresponding viability loss (*y*-axis). Viability loss was calculated as the absolute difference in percentage points (pp) between collection (T0) and delayed post-thaw viability (T2). Linear regression analysis demonstrated a statistically significant association between storage duration at −80 °C and viability loss (R^2^ = 0.28, *p* < 0.001). The red line indicates the regression fit, and the shaded area denotes the 95% confidence interval.

Linear regression demonstrated a statistically significant association between storage duration and post-thaw viability decline (R^2^ = 0.283, *p* < 0.001), with an estimated loss of 1.02% per 100 days (Figure 3):

$$Viability\;loss\;\left(\%\right)=0.01018\times Storage\;duration\;\left(days\right)-3.53$$

This suggests a time-dependent degradation pattern under −80 °C storage conditions, with moderate predictive strength.

**Figure 4:**
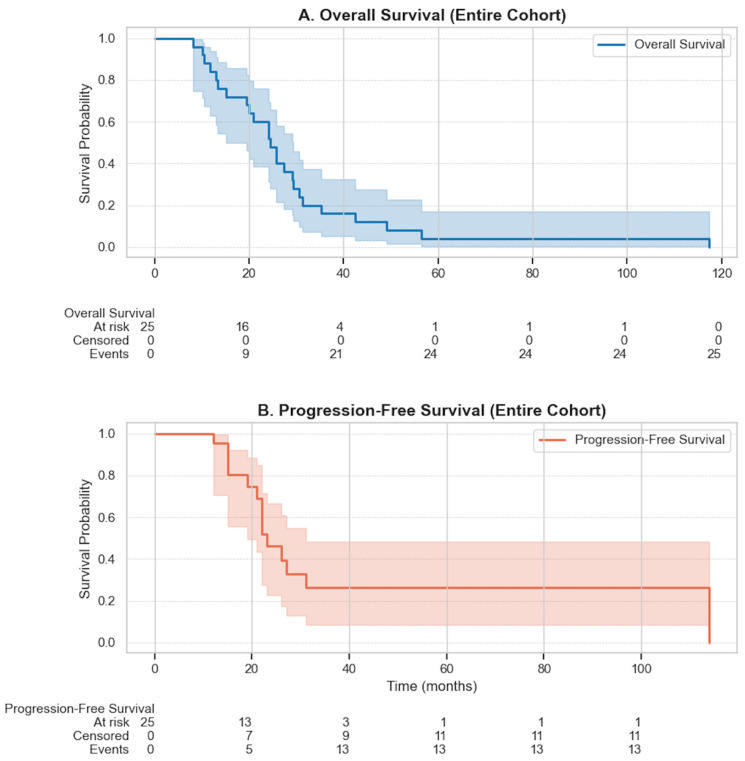
Overall and Progression-Free Survival in the Study Cohort. (**A**) Kaplan–Meier curve showing overall survival (OS) for all patients (n = 25), with a median OS of 24.5 months (95% CI: 19.5–29.3). (**B**) Kaplan–Meier curve for progression-free survival (PFS), with a median PFS of 23.0 months (95% CI: 19.0–31.0). Shaded areas represent 95% confidence intervals. No censoring was observed for OS, while 7 patients were censored in the PFS analysis.

Median OS was 24.5 months (95% CI: 19.5–29.3), and median PFS was 23.0 months (95% CI: 19.0–31.0) (Figure 4).

Viability loss between initial collection (T0) and post-thaw (T2) was assessed by both AO staining and flow cytometry. In the overall cohort, the mean viability loss from initial collection (T0) to post-thaw assessment (T2) was 9.2% with AO and 6.6% with flow cytometry. Among transplanted patients, the mean decline from collection to infusion (T0 to T1) was lower, at 3.2% (AO) and 2.8% (flow cytometry), indicating relatively limited viability loss during short-term storage. The Wilcoxon test demonstrated a significant difference in mean viability between AO and flow cytometry at T2 (*p* < 0.001), suggesting AO may be more sensitive in detecting delayed viability decline.

**Figure 5:**
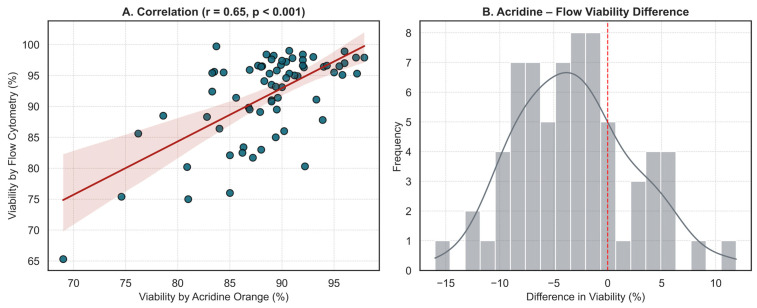
Agreement Between Acridine Orange and Flow Cytometry for Post-Thaw Viability Assessment. (**A**) Scatter plot showing a moderate and statistically significant correlation between viability measurements by acridine orange staining and flow cytometry (Pearson’s r = 0.65, *p* < 0.001). Each dot represents an individual paired measurement, and the solid red line represents the linear regression fit. The shaded area represents the 95% confidence interval of the regression line. (**B**) Histogram of paired differences between acridine orange and flow cytometry viability values. The red dashed line indicates the zero reference, representing no difference between methods. The distribution centers are near zero, indicating no systematic bias between methods. However, a slight negative skew suggests acridine orange may slightly overestimate viability relative to flow cytometry.

Finally, a comparison of the two measurement techniques in 69 samples revealed a moderate but significant correlation (r = 0.648, *p* < 0.001), indicating a consistent trend in viability estimation. The distribution of differences between the two methods was centered near zero, suggesting the absence of systematic measurement bias. Together, these findings support the complementary use of both methods in evaluating post-thaw cell quality, although flow cytometry may be more suitable for precise quantification in borderline cases (Figure 5).

## 4. Discussion

This single-center study systematically assessed the long-term viability and clinical efficacy of CD34^+^ progenitor cell products stored at −80 °C. Despite moderate viability loss over time, our findings demonstrate that most products retained sufficient functionality to ensure effective engraftment, reinforcing the viability of −80 °C as a practical alternative for cryopreservation in resource-limited settings.

The median post-thaw viability was 94.8%, and linear regression revealed a time-dependent decline (~1.02% per 100 days; R^2^ = 0.283, *p* < 0.001). These results are in line with prior studies showing stable CD34^+^ viability and successful transplantation even after extended −80 °C storage up to 6.6–15.8 years [2,20].

Clinically, hematopoietic recovery was achieved in nearly all patients, and neither storage duration nor donor age showed a significant correlation with CD34^+^ cell viability or dose. Reporting absolute CD34^+^ numbers before and after thawing—rather than viability percentages alone—is critical, since the clinically effective dose depends on the absolute viable CD34^+^ yield post-thaw. Providing both metrics ensures transparent evaluation of product quality and allows a more accurate interpretation of engraftment outcomes. Our findings confirm that product quality remains largely preserved under −80 °C conditions, consistent with long-term murine engraftment studies using products cryopreserved for up to 18 years [12].

However, disease biology clearly influenced engraftment outcomes. Patients with acute lymphoblastic leukemia had significantly prolonged neutropenia and delayed neutrophil recovery compared to multiple myeloma cases. These patterns are supported by prior studies reporting slower engraftment in patients with acute myeloid leukemia due to intensive pre-conditioning regimens and marrow suppression [21,22].

Interestingly, younger age was associated with significantly delayed neutrophil recovery in our cohort. However, this association likely reflects underlying disease distribution rather than a direct effect of age. Similar observations have been reported in pediatric cohorts, where younger children—particularly those with neuroblastoma—exhibited slower neutrophil recovery due to diagnosis-specific factors rather than chronological age itself [23]. Likewise, in adult populations, age initially appeared to influence engraftment kinetics, but its significance diminished in multivariate analysis when diagnosis and conditioning intensity were accounted for, suggesting that disease characteristics play a more decisive role than age alone [24].

In contrast to prior studies demonstrating that patients in complete remission tend to achieve faster platelet engraftment [25], our cohort revealed a significantly shorter time to platelet recovery among patients not in complete remission at the time of transplantation. While unexpected, this discrepancy may be attributed to disease-specific transplant dynamics or residual hematopoietic activity in partially treated marrow. Further studies are warranted to clarify whether this pattern is replicable across larger and more diverse cohorts.

Contrary to traditional assumptions favoring fresh grafts, we observed a significantly longer neutropenia duration and delayed neutrophil engraftment in recipients of fresh products. While this may appear unexpected, recent evidence suggests that cryopreserved grafts can perform comparably to fresh ones in specific clinical settings, particularly when logistical or donor constraints exist. For instance, Ersal et al. reported no significant difference in neutrophil engraftment between fresh and frozen grafts in matched sibling donors, while Keyzner et al. observed a delay in absolute neutrophil count recovery among cryopreserved graft recipients that was mitigated in fully matched cohorts [26,27]. These findings reinforce the idea that engraftment kinetics are shaped by multiple factors, including conditioning intensity, disease type, and graft source, rather than graft freshness alone.

Finally, our comparison of viability measurement techniques revealed a significant difference between AO staining and 7-AAD-based flow cytometry, with AO detecting greater viability loss in delayed post-thaw assessments. This aligns with the findings of Cai et al. [7], who reported that acridine orange/propidium iodide assays consistently yielded lower viability values—particularly in cryopreserved products—and emphasized the increased variability among assays under these conditions. Their study underscores the importance of careful assay selection for post-thaw viability evaluation, supporting our use of complementary methods to improve reliability in borderline cases. Additionally, Varan et al. found that the AO/EB staining method demonstrated the highest concordance with 7-AAD-based flow cytometry in assessing post-thaw viability, with an intraclass correlation coefficient of 0.72 (*p* < 0.05). Their findings suggest that AO/EB may more effectively detect subtle declines in viability, particularly in complex cell mixtures such as peripheral blood stem cell grafts, due to its reduced susceptibility to interference from erythrocytes and debris [4]. With respect to viability, both AO and flow cytometry primarily assess indirect markers of mitochondrial health (e.g., membrane integrity, nucleic acid stability, and metabolic activity), but they do not directly evaluate mitochondrial quality processes such as mitophagy, biogenesis, or mitochondrial dynamics. This clarification ensures that our data are interpreted within the appropriate biological context, reflecting mitochondrial health rather than comprehensive mitochondrial quality [28,29,30].

Liquid nitrogen (–196 °C) has long been considered the gold standard for cryopreservation of CD34^+^ progenitor cells, ensuring high viability and preserved engraftment capacity over extended storage [31]. However, this approach is limited by high costs, infrastructure requirements, and potential contamination risks [32]. In parallel, increasing evidence demonstrates that −80 °C mechanical freezer storage, particularly when combined with cryoprotectants such as DMSO and hydroxyethyl starch, can also maintain CD34^+^ cell viability above 75–80%, preserve clonogenic potential, and support successful hematopoietic reconstitution [2,20]. Temporary storage studies further indicate that CD34^+^ cell integrity is preserved for several weeks at −80 °C in case of liquid nitrogen system failures [33]. Taken together, both liquid nitrogen and −80 °C cryopreservation are effective for maintaining CD34^+^ progenitor cell functionality, with liquid nitrogen remaining the established standard and −80 °C offering a practical, safe, and cost-effective alternative in resource-limited settings.

While this study is limited by its single-center design and modest cohort size, the consistent viability assessments across 72 long-term cryopreserved products and their correlation with clinical engraftment outcomes provide strong real-world evidence supporting the reliability of −80 °C storage. However, the absence of functional assays, such as colony-forming unit tests or in vivo engraftment models, limits definitive conclusions regarding long-term hematopoietic capacity. Future multicenter and prospective studies integrating functional endpoints are warranted to validate these findings and refine viability thresholds for clinical decision-making.

## 5. Conclusions

This study highlights the clinical feasibility of long-term CD34^+^ progenitor cell cryopreservation at −80 °C, demonstrating that most products retain sufficient viability to support successful engraftment despite time-dependent decline. Transplantation outcomes were more closely associated with disease biology and remission status than with post-thaw viability alone. Importantly, our viability-loss model offers a practical framework for anticipating product quality under extended storage conditions. AO staining showed superior sensitivity in detecting delayed cellular injury, likely reflecting early membrane disruption or mitochondrial health not captured by flow cytometry. Its complementary use may be particularly informative when evaluating marginal-quality or long-stored grafts. These findings offer a basis for refining product evaluation strategies and may inform graft selection and clinical decision-making, especially in real-world or resource-constrained transplant settings.

## Figures and Tables

**Table 1:** Baseline Demographic and Clinical Characteristics of the Study Population (N = 25).

Characteristic	n (%) or Median (Range)
**Gender**	
Female	10 (40%)
Male	15 (60%)
**Age (years)**	54 (19–63)
**Body Mass Index (kg/m^2^)**	25.9 (18.6–38.3)
**Diagnosis**	
Hodgkin lymphoma (HL)	1 (4%)
Non-Hodgkin lymphoma (NHL)	2 (8%)
Multiple myeloma (MM)	13 (52%)
Acute lymphoblastic leukemia (ALL)	8 (32%)
Chronic myelomonocytic leukemia (CMML)	1 (4%)
**Mobilization Regimen**	
Unstimulated bone marrow	1 (4%)
G-CSF	19 (76%)
Etoposide + G-CSF	4 (16%)
Plerixafor + G-CSF	1 (4%)
**Transplantation Status**	
Transplant performed	20 (80%)
Not performed	5 (20%)
Conditioning Regimen and Intensity *	
Melphalan 200 mg/m^2^ (Mel-200)	10 (50%)
Melphalan 140 mg/m^2^ (Mel-140)	2 (10%)
Busulfan + Cyclophosphamide (Bu-Cy)	5 (25%)
Etoposide + Cyclophosphamide + TBI	3 (15%)
Myeloablative	20 (100%)
**Radiotherapy**	3 (12%)
**Number of Prior Treatment Lines**	
1 line	10 (40%)
2 lines	8 (32%)
3 lines	4 (16%)
4 lines	1 (4%)
5 lines	2 (8%)
**Neutrophil engraftment > 0.5 × 10^9^/L (days)**	13.5 (7–23)
**Platelet engraftment > 20 × 10^9^/L (days)**	13.5 (7–30)
**Platelet engraftment > 50 × 10^9^/L (days)**	16.5 (13–37)

* Conditioning regimen and intensity were evaluated only among patients who underwent stem cell infusion (n = 20). Abbreviations: G-CSF, Granulocyte colony-stimulating factor; TBI, Total body irradiation.

**Table 2:** Characteristics of All Collected Stem Cell Products (N = 72 Products).

Parameter	Median (Range)
Storage duration at −80 °C (days)	868 (382–1763)
Total CD34^+^ cells collected (µL)	1770.5 (18–3591)
Viable CD34^+^ cells collected (µL)	1242 (18–3591)
Total cell viability (%)	100 (95.2–100)
CD34^+^ cell viability (%)	100 (98.6–100)
CD34^+^ cell dose (×10^6^/kg recipient body weight)	8.5 (3.3–14.0)
Harvest WBC count (×10^9^/L)	180.5 (45.3–381.0)
Harvest neutrophil count (×10^9^/L)	72.9 (0.01–263.0)
Harvest lymphocyte count (×10^9^/L)	51.4 (8.97–191.0)
Harvest monocyte count (×10^9^/L)	27.9 (7.58–135.0)
Mononuclear cell to total WBC ratio	0.60 (0.23–0.99)

Abbreviations: WBC, White Blood Cell.

**Table 3:** Comparison of CD34^+^ Cell Dose and Engraftment Parameters by Clinical Subgroups.

Characteristic	CD34^+^ Cell Dose (×10^6^/kg)	*p*	Neutropenia Duration (Days)	*p*	ANC > 0.5 × 10^9^/L (Days)	*p*	PLT > 20 × 10^9^/L (Days)	*p*	PLT > 50 × 10^9^/L (Days)	*p*
**Gender**		0.7868		0.786		0.6692		0.9623		1.000
Female	5.4 (4.2–6.7)		11 (7–36)		13.5 (11–23)		13 (10–21)		16.5 (13–36)	
Male	6.0 (3.4–8.1)		14 (6–27)		13.5 (7–23)		13.5 (7–30)		17.5 (13–37)	
**Comorbidity**		0.8364		0.2809		0.0967		0.4557		0.4941
Absent	6.0 (3.5–8.1)		27 (6–27)		19.5 (11–23)		12 (11–17)		16 (13–26)	
Present	5.4 (3.4–6.8)		10 (6–36)		12.5 (7–23)		14 (7–30)		17 (13–37)	
**Diagnosis**		0.105		<0.001		<0.001		0.501		0.558
Multiple Myeloma	4.8 (3.4–6.8)		8.5 (6–18)		11 (7–19)		12.5 (7–30)		17 (13–37)	
Acute Lymphoblastic Leukemia	6.0 (4.2–8.1)		27 (12–36)		19.5 (15–23)		14.5 (11–21)		16 (13–30)	
**CR Status**		0.636		0.296		0.341		0.012		0.161
Yes	5.9 (3.4–6.8)		14.5 (6–36)		14.5 (10–23)		14.5 (10–30)		19 (13–37)	
No	4.8 (3.5–8.1)		8.5 (6–27)		11.5 (7–22)		11 (7–12)		15.5 (13–16)	
**Fresh Product**		0.1483		0.0144		0.0179		0.5708		0.2790
Yes	6.0 (5.9–6.1)		27 (12–36)		17 (15–23)		14.5 (12–17)		14 (13–26)	
No	4.8 (3.4–8.1)		9 (6–28)		11.5 (7–23)		12.5 (7–30)		17 (13–37)	

Abbreviations: CR, Complete Response; ANC, Absolute Neutrophil Count; PLT, Platelet Count.

**Table 4:** Comparison of Product and Harvest Characteristics Between Frozen and Fresh Stem Cell Grafts in Transplanted Patients.

Parameter	Frozen Group (Patients, n = 6), Median (Range)	Fresh Group (Patients, n = 14), Median (Range)	*p*
Total CD34^+^ cells collected (µL)	2870.0 (45.0–3591.0)	2050.0 (18.0–2700.0)	0.2857
Viable CD34^+^ cells collected (µL)	2866.0 (45.0–3591.0)	1368.0 (18.0–2685.0)	0.1773
Total cell viability (%)	96.2 (95.7–96.8)	96.5 (93.5–96.9)	0.7110
CD34^+^ cell viability (%)	100.0 (99.7–100.0)	100.0 (98.6–100.0)	0.8135
CD34^+^ cell dose (×10^6^/kg recipient body weight)	6.0 (5.9–6.1)	4.8 (3.42–8.1)	0.1483
Harvest WBC count (×10^9^/L)	237.0 (65.5–314.0)	171.9 (45.3–381.0)	0.3425
Harvest neutrophil count (×10^9^/L)	119.5 (0.01–263.0)	61.8 (0.02–102.0)	0.0326
Harvest lymphocyte count (×10^9^/L)	79.9 (51.4–137.0)	53.9 (22.8–191.0)	0.1297
Harvest monocyte count (×10^9^/L)	46.4 (12.2–86.0)	64.1 (8.3–135.0)	0.3530
Mononuclear cell to total WBC ratio	0.60 (0.37–0.99)	0.61 (0.47–0.99)	0.7103

Abbreviations: WBC, White Blood Cell.

**Table 5:** Spearman Correlation Between Patient Age and Transplant-Related Outcomes.

Parameter	Correlation Coefficient (ρ)	*p*-Value	N
Post-thaw viability (Flow cytometry)	−0.087	0.6784	25
CD34^+^ cell viability (%)	−0.181	0.3877	25
Total cell viability (%)	0.176	0.5853	25
CD34^+^ cell dose (×10^6^/kg recipient body weight)	−0.342	0.14	20
Neutropenia duration (days)	−0.761	<0.001	20
ANC > 0.5 × 10^9^/L (days)	−0.75	<0.001	20
PLT > 20 × 10^9^/L (days)	−0.022	0.931	18
PLT > 50 × 10^9^/L (days)	0.066	0.808	16

Abbreviations: ANC, Absolute Neutrophil Count; PLT, Platelet Count.

## Data Availability

Data is available on request from the authors.

## Abbreviations

AO	acridine orange
ALL	acute lymphoblastic leukemia
ANC	absolute neutrophil count
CD34^+^	cluster of differentiation 34 positive
CR	complete remission
DMSO	dimethyl sulfoxide
EB	ethidium bromide
ELN	European LeukemiaNet
G-CSF	granulocyte-colony stimulating factor
HL	Hodgkin lymphoma
HSC	hematopoietic stem cell
MM	multiple myeloma
NHL	non-Hodgkin lymphoma
OS	overall survival
PFS	progression-free survival
PLT	platelet
TBI	total body irradiation
T0	initial collection time point
T1	pre-infusion time point
T2	delayed post-thaw assessment time point
WBC	white blood cell
7-AAD	7-aminoactinomycin D
